# Alum and a TLR7 agonist combined with built-in TLR4 and 5 agonists synergistically enhance immune responses against HPV RG1 epitope

**DOI:** 10.1038/s41598-023-43965-3

**Published:** 2023-10-05

**Authors:** Maryam Mashhadi Abolghasem Shirazi, Seyed Mehdi Sadat, Setareh Haghighat, Farzin Roohvand, Arash Arashkia

**Affiliations:** 1 Department of Microbiology, Faculty of Advanced Sciences and Technology, Tehran Medical Sciences, Islamic Azad University, Tehran, Iran; 2 Department of Molecular Virology, Pasteur Institute of Iran, No. 69, Pasteur Ave, Tehran, Iran; 3 Department of Hepatitis, AIDS and Blood borne Diseases, Pasteur Institute of Iran, Tehran, Iran

**Keywords:** Biotechnology, Cancer, Computational biology and bioinformatics, Immunology, Microbiology

## Abstract

To relieve the limitations of the human papillomavirus (HPV) vaccines based on L1 capsid protein, vaccine formulations based on RG1 epitope of HPV L2 using various built-in adjuvants are under study. Herein, we describe design and construction of a rejoined peptide (RP) harboring HPV16 RG1 epitope fused to TLR4/5 agonists and a tetanus toxoid epitope, which were linked by the (GGGS)_3_ linker in tandem. In silico analyses indicated the proper physicochemical, immunogenic and safety profile of the RP. Docking analyses on predicted 3D model suggested the effective interaction of TLR4/5 agonists within RP with their corresponding TLRs. Expressing the 1206 bp RP-coding DNA in *E. coli* produced a 46 kDa protein, and immunization of mice by natively-purified RP in different adjuvant formulations indicated the crucial role of the built-in adjuvants for induction of anti-RG1 responses that could be further enhanced by combination of TLR7 agonist/alum adjuvants. While the TLR4/5 agonists contributed in the elicitation of the Th2-polarized immune responses, combination with TLR7 agonist changed the polarization to the balanced Th1/Th2 immune responses. Indeed, RP + TLR7 agonist/alum adjuvants induced the strongest immune responses that could efficiently neutralize the HPV pseudoviruses, and thus might be a promising formulation for an inexpensive and cross-reactive HPV vaccine.

## Introduction

Human papillomaviruses (HPV) are a heterogeneous group of around 200 types that cause diseases ranging from benign epithelial warts to life-threatening anogenital tract and oropharynx cancers. It is estimated that around 50% and 20% of global cervical carcinomas are caused by HPV type 16 and 18, respectively (reviewed in Ref.^1^).

To date, six licensed/prequalified virus-like particle (VLP)-based vaccines against HPV infection are available that induce type-specific neutralizing antibody (nAb) against major capsid protein (L1) of the virus. They include: three bivalent vaccines (Cecolin/Cervarix/Walrinvax) containing HPV16/18 L1 VLPs, two quadrivalent (Cervavac/Gardasil) and one nonavalent vaccine (Gardasil9) that besides HPV16/18 L1 VLPs, contain that of the other pathogenic HPVs (reviewed in Ref.^2^). However, despite the efficacy of the HPVL1-based vaccines, several limitations such as the type-specificity (limited cross-protections), high cost and technical complexity of the VLP production process, restrict their versatile utilization (reviewed in Ref.^3^).

Besides dominant and type-specific conformational HPV L1 epitopes that induce high titers of protective nAbs, the N-terminal region of HPV minor capsid protein (L2) that is not able to form VLP, contains several conserved pan-type linear epitopes. These epitopes have the potentiality of eliciting broadly cross-reactive nAbs against different HPV genotypes, even in the isolated forms, albeit in much lower titers and potency compared to the L1-based VLP reviewed in Refs.^3,4^). Among HPV L2 conserved epitopes, the 17–36 epitope (initially identified by the mouse monoclonal Antibody “RG1”) is one of the most studied epitopes with a high potency of inducing cross-nAbs but in low titers^5^. In this context, various strategies were studied to increase the immunogenicity of HPV L2 RG1 epitope. One strategy was using HPV L1 capsid as a scaffold to make L2-presenting VLPs or displaying the RG1 epitope on various bacteriophages and viruses (reviewed in Ref.^4,5^). But these approaches were again complex processes that would result in the high-cost production. Consequently, approaches to increase the immunogenicity of the RG1 epitope while reducing the cost of production were introduced. These approaches were mainly focused on the bacterial expression of tandem (head to tail) repeats (three to six) of the RG1 multi-epitopes^5–7^ either alone or fused with built-in adjuvants^8^ and/or subsequent formulation (combination) of the purified bacteria-derived antigen (Ag) with classic adjuvants (reviewed in Refs.^3,9^).

It has been shown that incorporation of a built-in adjuvant in fusion with the epitopic antigen might induce potent innate immune responses to stimulate antigen-specific acquired immunity^8^. Built-in adjuvants usually consist of bacterial toxoids and agonists of pattern recognition receptor ligands such as toll-like receptors (TLRs)^8^. Tetanus toxoid (TT) is an example of a bacterial toxoid that has multiple CD4^+^ epitopes and can act as a universal helper T cell (Th) epitope.

Application of the P2 epitope of TT (TT-P2; consisting of 15 amino acids) as a small and effective built-in adjuvant for a multi-epitopic vaccine against foot-and-mouth disease virus was recently reported^10^. In parallel, due to their easier accessibility, cell-surface TLRs might be preferably selected for being targeted by built-in TLR agonists.

For example, the bacterial flagellin engages cell surface TLR5 (an innate immunity receptor) that triggers the subsequent signaling pathways to bridge between innate and adaptive immunity^11^. It has been reported that fusion of bacterial flagellin to either HPV L2 11–200 or multimers of 11–88 peptides (without the use of any exogenous adjuvant) raised strong cross-protective nAbs in immunized mice^12^. Interestingly, both the N- and C-termini of flagellin function as a TLR5 agonist even in the absence of the highly antigenic region between the two end termini. Therefore, this middle antigenic region can be replaced by target Ags and epitopes to restore TLR5 agonist characteristics while disabling the ability of the host to produce antibodies against bacterial flagellin^8,13^. Application of this strategy for in silico design of an HPV16 L2-based prophylactic peptide vaccine candidates harboring two epitopes (10–36 and 65–89) was previously reported^7,14^. Currently, a recombinant derivative of flagellin with reduced immunogenicity (Entolimod or CBLB502), is under clinical trial as a therapeutics in patients with advanced cancers (ClinicalTrials.gov Identifier: NCT01527136) to suppress TLR5-expressing tumors^15^. Similarly, RS09, an LPS mimetic short synthetic peptide (APPHALS) that is a TLR4 agonist was shown to promote NF-κB nuclear translocation to induce inflammatory cytokine secretion in macrophages^16^. Recently, the successful application of RS09 as a built-in adjuvant for the construction of vaccine candidates against several infectious diseases, such as SARS-CoV-2^17^ and malaria^18^ has been reported. In addition, in silico design and computational analysis of an immunogen harboring two overlapped epitopes of HPV58 L2 protein fused with flagellin and RS09 showed the stability and solubility of the construct^19^. More recently, immunization of mice by two epitopic domains (10–36 and 65–89) of HPVL2 fused to flagellin, RS09 and two universal T-helper epitopes was studied. Although preliminary results of this study indicated overall induction of humoral and cellular responses but no specific immune tests were addressed^20^. There are accumulating evidences that the proper combination of several built-in adjuvants (especially TLR agonists) alone or in formulation with external adjuvants might synergistically boost the immune responses against the targeted antigenic peptide^21,22^. In this context, the synergistic effect of combined use of alum and monophosphoryl lipid A (MPL as a TLR4 agonist) for a multi-type HPV L2 fusion peptide^21^, and also TLR7 agonist either as external adjuvant (AS37) for a subunit SARS-CoV-2 vaccine^23^ or as a built-in adjuvant for an anti-tumor vaccine^22^ to enhance the immune responses have been shown.

Several prior studies have already reported the application of various built-in and external adjuvants to enhance immune responses against HPV L2 peptide vaccine candidates; however, they were restricted to using either other epitopic peptides rather than RG1^12,19^, or a single built-in adjuvant^7,12^, or only in silico analyses without any specific in vivo validation studies^14,19^. Moreover, to our knowledge, the synergistic effects of the combined use of several built-in components in addition to the use of external adjuvants to provide the maximum enhancement of the multi-RG1 immunogen-peptides were not reported before. To address these concerns, we conducted the present study as a “exploratory phase to proof the concept” and assessed by both in silico and in vivo analyses, the critical role of a properly designed built-in adjuvant harboring TLR 4/5 agonists + TT-P2 on anti-RG1 immune responses. In addition, the synergistic effect of a TLR7 agonist and/or alum adjuvants (alone or combined) to enhance the anti-HPV RG1 immune responses within the designed built-in was studied.

## Results

### In silico analyses

#### Analyses indicated the proper physicochemical, immunogenicity and safety characteristics of the RP (rejoined immunogenic-peptide: “d3RG-T5-TT-T4”)

As summarized in Table 1, in silico analyses predicted that the RP is stable, polar, hydrophilic with a positive charge, non-allergen, non-toxic antigen, and soluble when expressed in *E. coli* (Table 1).

**Table 1:** Physicochemical, immunogenicity and safety characteristics of the RP predicated by in silico analyses.

No.	Evaluated characteristics	Results
1	Molecular weight	43,183.96 kDa
2	Instability index	35.84^A^
3	Gravy	− 0.449^B^
4	Aliphatic index	69.63
5	Theoretical pI	8.36^C^
6	Total no. of negatively charged residues (Asp + Glu)	35
7	Total no. of positively charged residues (Arg + Lys)	39
8	No. of amino acids	433 aa
9	Antigenicity (Vaxijen v.2)*	Probable antigen (1.4032)^D^
10	Solubility (Solpro)*	0.900941 Soluble^E^
11	Solubility (ccSol)*	100% Soluble^F^
12	Allergenic characteristics (AllergenFP)*	Probable non-allergen (index 0.82)^G^
13	Toxicity (Toxinpred)*	Non-toxic

^A^If the instability index is below 40, the protein is stable. Therefore, index instability of 35.84 indicates the stability of the RP.

^B^“Gravy” predicts the hydrophilicity/hydrophobicity of the protein. More negative values indicate more hydrophilicities. Therefore, Gravy of -0.449 indicates that RP is hydrophilic.

^C^pH in which the protein does not have a specific surface electric charge. Therefore, pI of 8.36 represents the isoelectric point of the RP.

^D^Antigenicity values above the threshold (0.4) indicate the antigenicity/immunogenicity of the protein. Therefore, antigenicity score of 1.4032 indicated that RP might be a proper antigen (immunogen).

^E^The solubility of the protein following overexpression in *E. coli* is measured. A score of 0.900941 indicates the solubility of RP.

^F^Based on the physicochemical properties of the amino acid sequence, the solubility of the RP was predicated to be 100%.

^G^The allergenic scores above 0.759 are considered as non-allergen. Therefore, allergenicity score of 0.82 indicated that RP might be non-allergen.

*The utilized softwares are indicated in parentheses (please see methods section for more details).

#### Results of the structural analyses of RP

**Figure 1:**
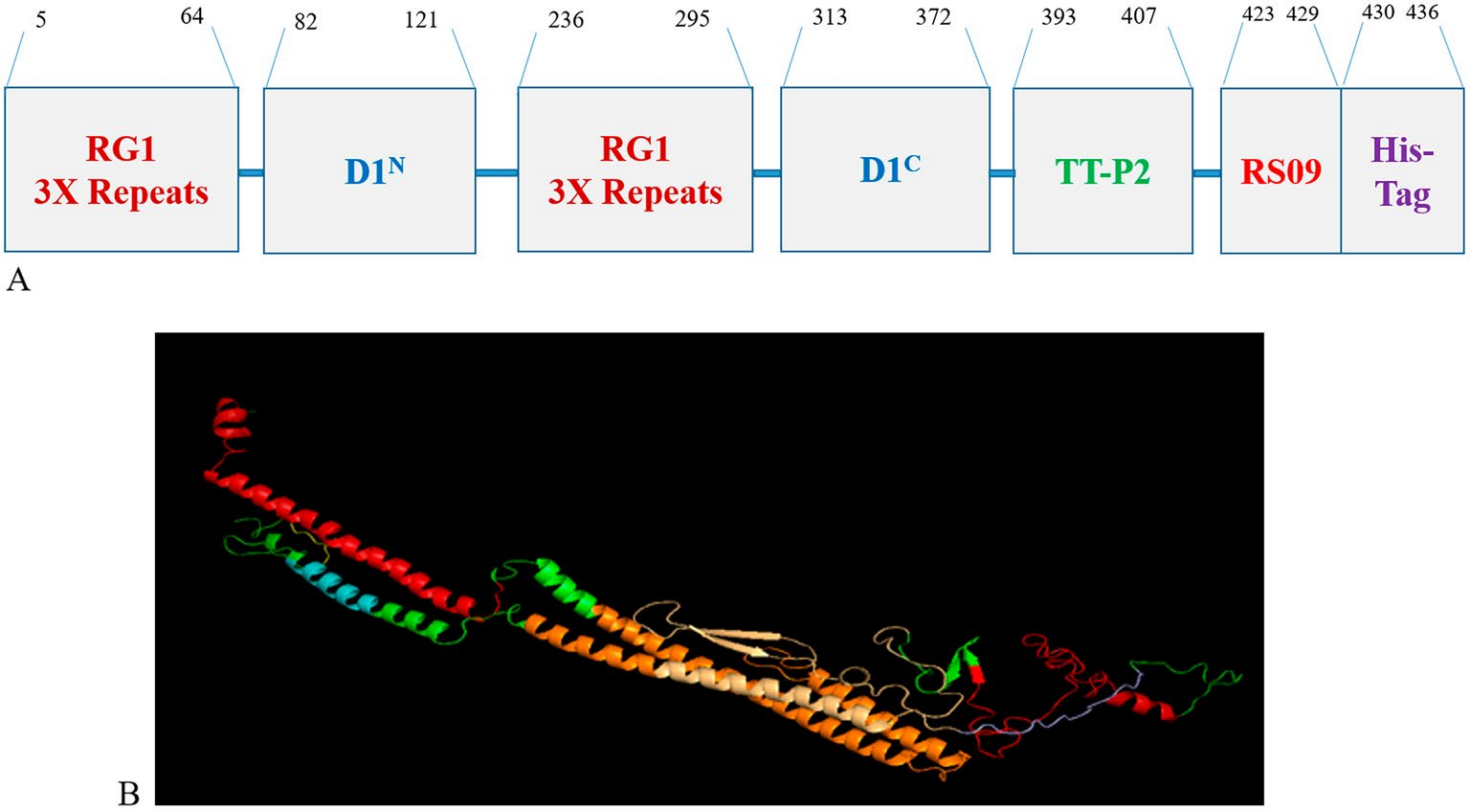
Schematic view and the refined 3D structure of the RP construct of the rejoined immunogenic-peptides (RP). (**A**) The Immunogenic RP contained the dual 3 × tandem repeats of the RG1 epitope and TLR5/4 built-in-adjuvants and TT-P2 “d3RG-T5-TT-T4” in tandem linked by short flexible linkers of (GGGS)_3_ presented as the blue dashes. The displayed numbers indicate the amino acid position of each fragment. The N- and C- termini of the D1 domain (D1^N^ and D1^C^) of flagellin (the TLR5 agonist), TT-P2: tetanus toxoid epitope (universal T-cell helper), RS09 (the short TLR4 agonist peptide), and His-tag (6xHis) for facilitating detection and purification of the antigen are shown. (**B**) The 3D structure of the RP was modeled by I-TASSER, and the best proposed model was refined by 3Drefine server. According to the results of 3drefine server, the 3D structure of the RP is optimal in terms of the network of hydrogen bonds and the amount of energy at the atomic level. RG-1 is shown in red, D1^N^ segment in orange, D1^C^ segment in light orang, linker in light blue, P2 in blue, RS09 in yellow, and (GGGS)_3_ linker in green.

As shown in the Fig. S1, secondary structure analyses by SOPMA showed that RP consisted of alpha-helix (35.10%), extended strand (16.63%), beta-turn (7.62%), and random coil (40.65%). The 3D structure of the RP by the I-TASSER server introduced five models, and the model with the highest C-score value (− 1.03) was selected for further analyses (Fig. 1).

To obtain a more chemically accurate model for high-resolution performance analysis, the I-TASSER-derived models were further refined with 3Drefine. Accordingly, five parameters, including GDT-TS, GDT-HA, RMSD, RWplus, and MolProbity, were optimized. The higher GDT-TS, RMSD and GDT-HA scores and the lower scores of 3Drefine, MolProbity, and RWplus showed a higher quality model. Based on these parameters, refined model No. 1 was selected for validation analyses (Table S2).

**Figure 2:**
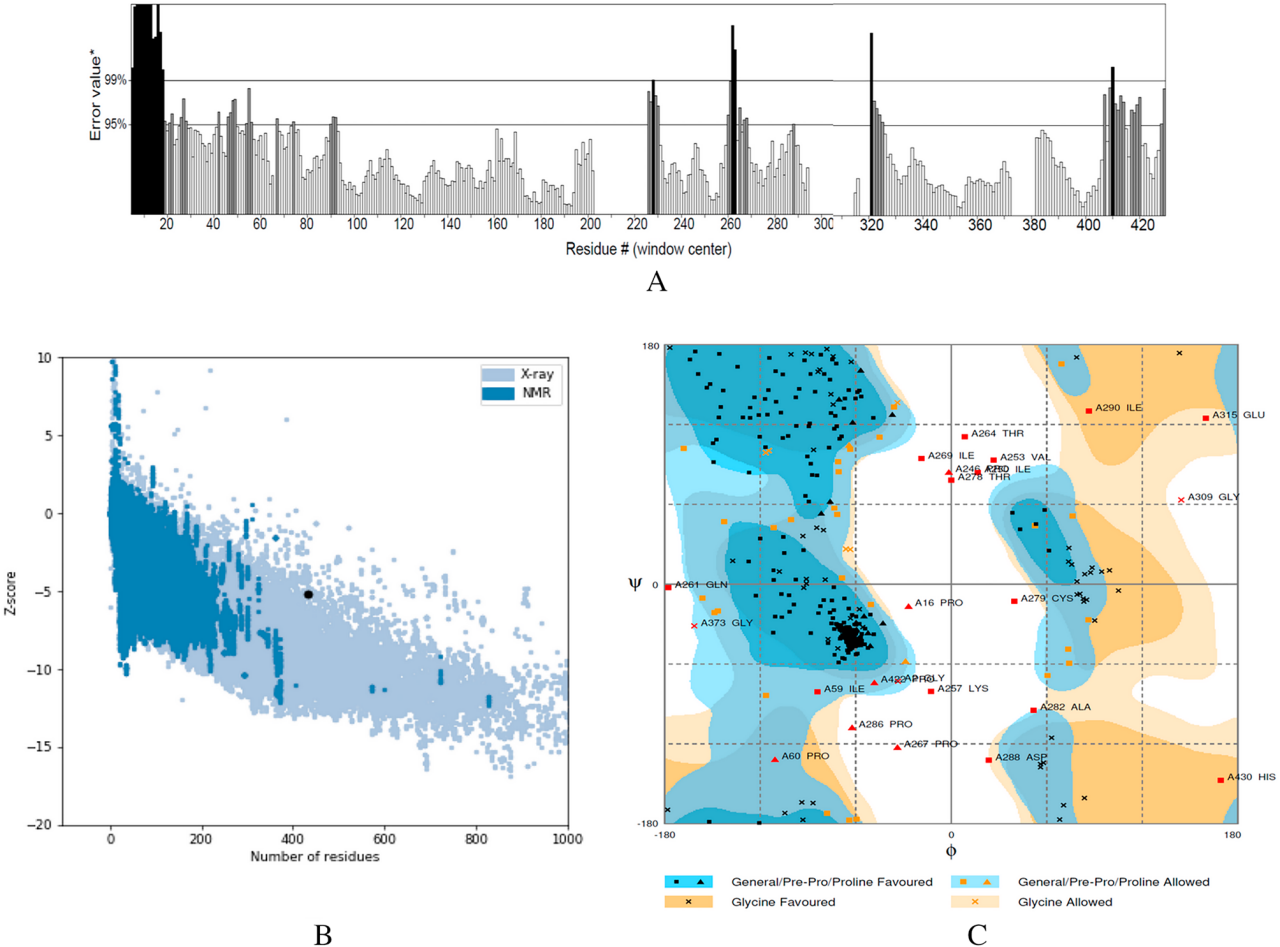
Validation results of the refined RD structure. (**A**) The ERRAT plot analysis showed that the overall quality factor of the refined construct is as high as 82.60%**. *In the error axis, two lines were drawn to ensure that areas that are higher than the error value can be passed. **Expressed as the percentage of the protein. The calculated error value falls below the 95% rejection threshold. High-resolution structures generally produce values of about 95% or higher. For low resolution (2.5 to 3 A), the average overall quality factor is about 91%. (**B**) ProSA-web z-score plot for refined model was -5.19 (indicating the overall quality). (**C**) The RAMPAGE plot analysis. In the Ramachandran plot of the refined model, the generally favored region, Pre–Pro and proline-favored regions are indicated in blue color. The generally allowed region, Pre–Pro and proline-allowed regions are shown in dark and pale blue. The Glycine-favored and allowed regions are shown in dark and pale orange. Overall validation results of the refined structure showed that only very few residues might be located in unauthorized areas, indicating the quality of the predicted structure.

The result of the model validation by the ERRAT plot showed that the overall quality factor of the refined RP construct was as high as 82.60%. (Fig. 2A). Based on the ProSA web plot, the Z-score of the RP was − 5.19 which indicated the overall model quality. Its value was displayed in a fragment that contained the Z-scores of all experimental protein chains determined in the refined PDB in Fig. 2B. Additionally, the Ramachandran plot obtained by RAMPAGE plot analysis showed that 86.8% of the residues were in the most favored region, 7.9% in the allowed region, and 5.4% were displayed in outlier regions (Fig. 2C). These results showed that only very few residues might be located in unauthorized areas, indicating the quality of the predicted structure.

#### Molecular docking analyses

**Figure 3:**
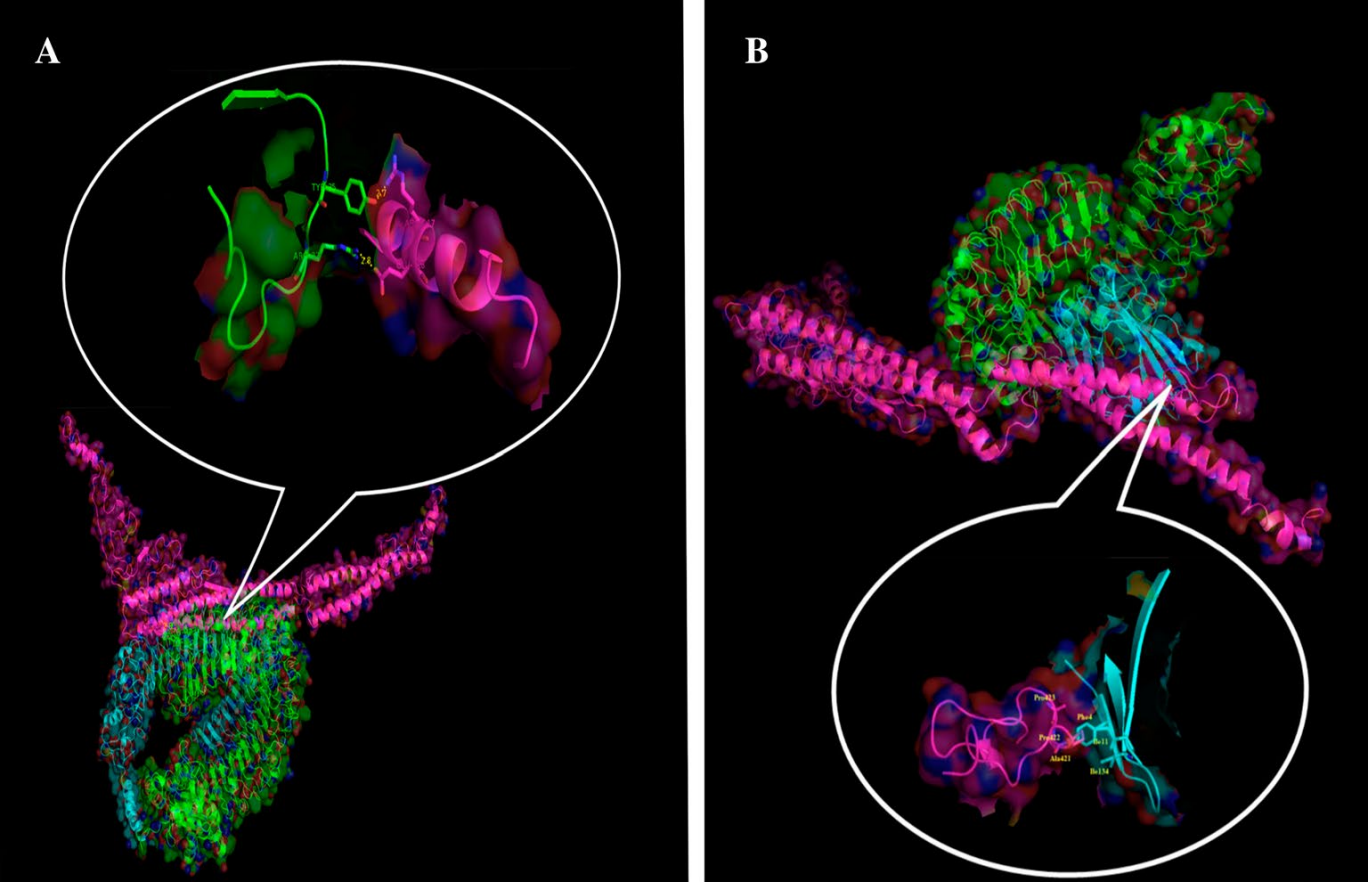
The 3D structure of molecular docking simulation between TLR4 and TLR5 with the corresponding ligand. (**A**) Molecular docking between TLR5 and the flagellin D1 domain in the RP construct. The pink chain is the RP construct and the blue and green chains are TLR5. (**B**) Molecular docking between TLR4/MD2 and the RS09 epitope in the RP construct. The pink chain is the RP construct, the blue chain is MD2, and the green chain is TLR4. Results of the interaction analyses of the RP with TLR4 and 5 (protein–protein docking) by SwarmDock server indicated good affinities between the receptor (TLR 4 and 5) and the modeled RP (TLR 4 and 5 agonists (highly conserved regions of D1 domain flagellin and and RS09, respectively) within modeled RP).

As shown in Fig. 3A and B, results of the analyses of the interaction of the RP with TLR4 and 5 (protein–protein docking) indicated high SwarmDock scores for TLR5-flagellin D1 domain interaction (E-total of − 33.21 kJ/mol), and TLR4/MD2-RS09 (E-total of − 27.10 kJ/mol), representing the good affinity between the receptor (TLR 4 and 5) and the modelled RP (TLR 4 and 5 agonists within modelled RP). Besides, the results of the molecular docking predicted that the TLR4/MD2 and TLR5 agonists in the RP construct could efficiently bind to their receptors (Fig. 3), which were similar to their natural counterparts with E-total of − 41.18 for TLR5-natural flagellin (Fig. S2A), and E-total of -29.63 for TLR4/MD2-intact RS09 (Fig. S2B) complexes.

**Figure 4:**
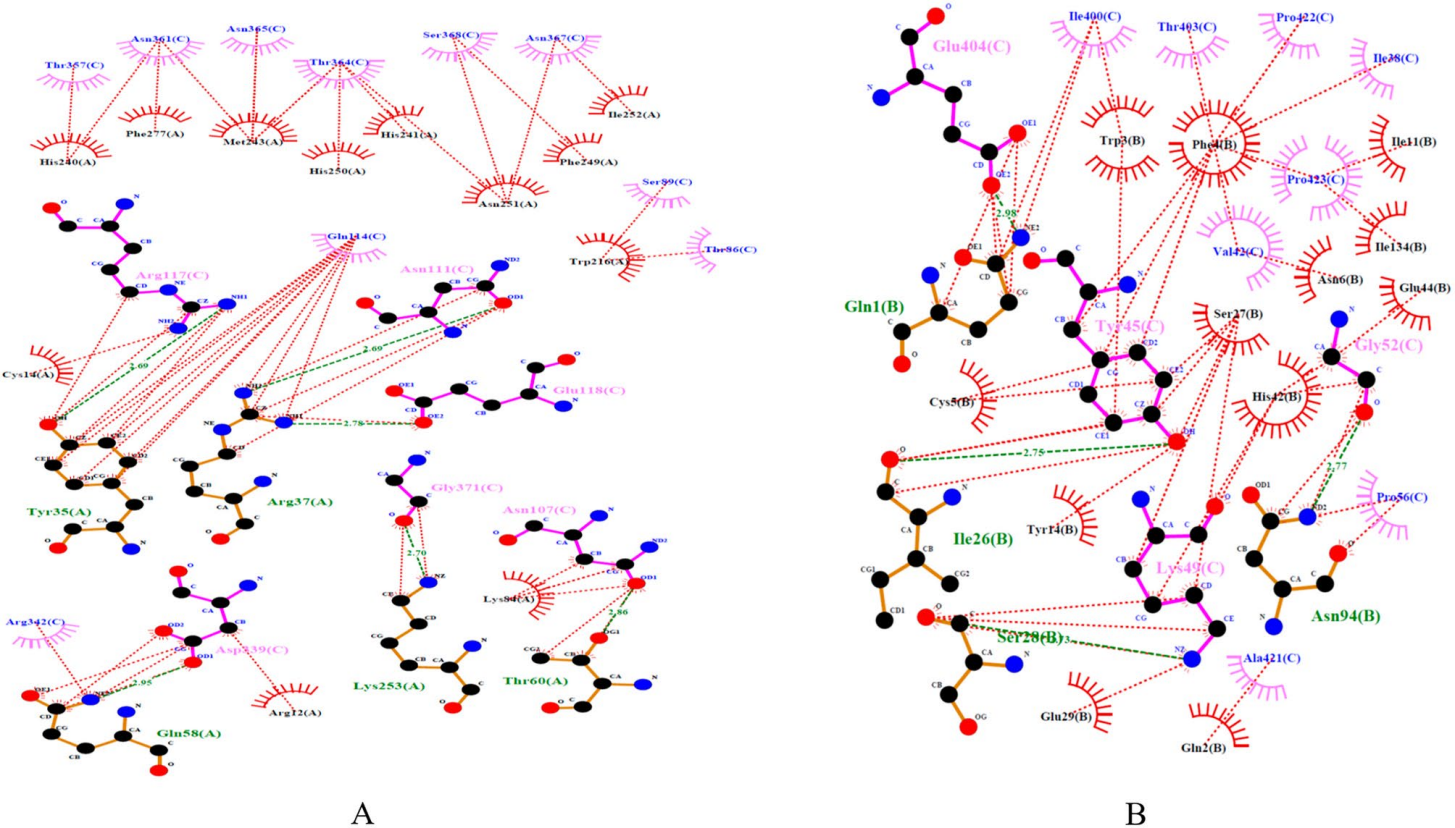
Analysis of the hydrogen bonds and hydrophobic interactions between the RP and TLR 4, 5 receptors. The green lines are hydrogen bonds and the red lines are hydrophobic interactions. (**A**) The interaction of Arg 117 and Glu 118 of the highly conserved region of the D1^N^ domain in the RP with TLR5 is shown. The hydrogen bond lengths are shorter than 2.95 Å. (**B**) Hydrophobic Interactions by Ala 421, Pro 422 and Pro 423 of RS09 in RP with MD-2/TLR4 receptor. The hydrogen bond lengths are shorter than 2.98 Å. Results of these analyses indicated the proper interactions of ligands RS09 and D1^N^ domain within RP to TLR4 and TLR5 receptors, respectively that might activate NFkB signaling pathway.

Finally, the hydrogen bonds and hydrophobic interactions between the RP construct and TLR5 (Fig. 4A), natural Salmonella flagellin and TLR5 (Fig. S2C), the RP construct and TLR4/MD2 (Fig. 4B), and the intact RS09 and TLR4/MD2 (Fig. S2D) were evaluated by Dimplot analysis. Results of these analyses indicated the proper interactions of RS09 and D1^N^ domain of flagellin in the RP with TLR4 and TLR5 receptors, respectively that were similar to their natural ligand interactions.

### In vitro analyses

#### Production and characterization of RP protein for immunization studies

The pET28a vector harboring 1206 bp RP-encoding DNA sequence (pET28a-RP) was confirmed by restriction analysis with *Nco*I-*Xho*I and *Xba*I-*Xho*I enzyme pairs (Fig. S3). Induction of *E.coli* BL21 (DE3) harboring pET28a-RP by IPTG resulted in the expression of recombinant protein with a molecular weight (MW) of 46 kDa (Fig. S4A). Western blot result confirmed the induction of the RP protein (Fig. S4B), and purification by Ni–NTA chromatography in native conditions resulted in a homogenous protein band corresponding to the confirmed protein band with yields around 0.7 mg/ml and purity of 96%. The solubility of the expressed protein was about 87% that was nearly in agreement with the results of the in silico analyses with Solpro and ccSol servers (Table 1). Additionally, evaluation of the endotoxin level of the purified RP indicated values less than 0.01 EU/ml, which was appropriate for the aim of the immunization.

#### Immunization by RP + TLR7 agonist/alum adjuvants induced the strongest anti-RG1 humoral responses

**Figure 5:**
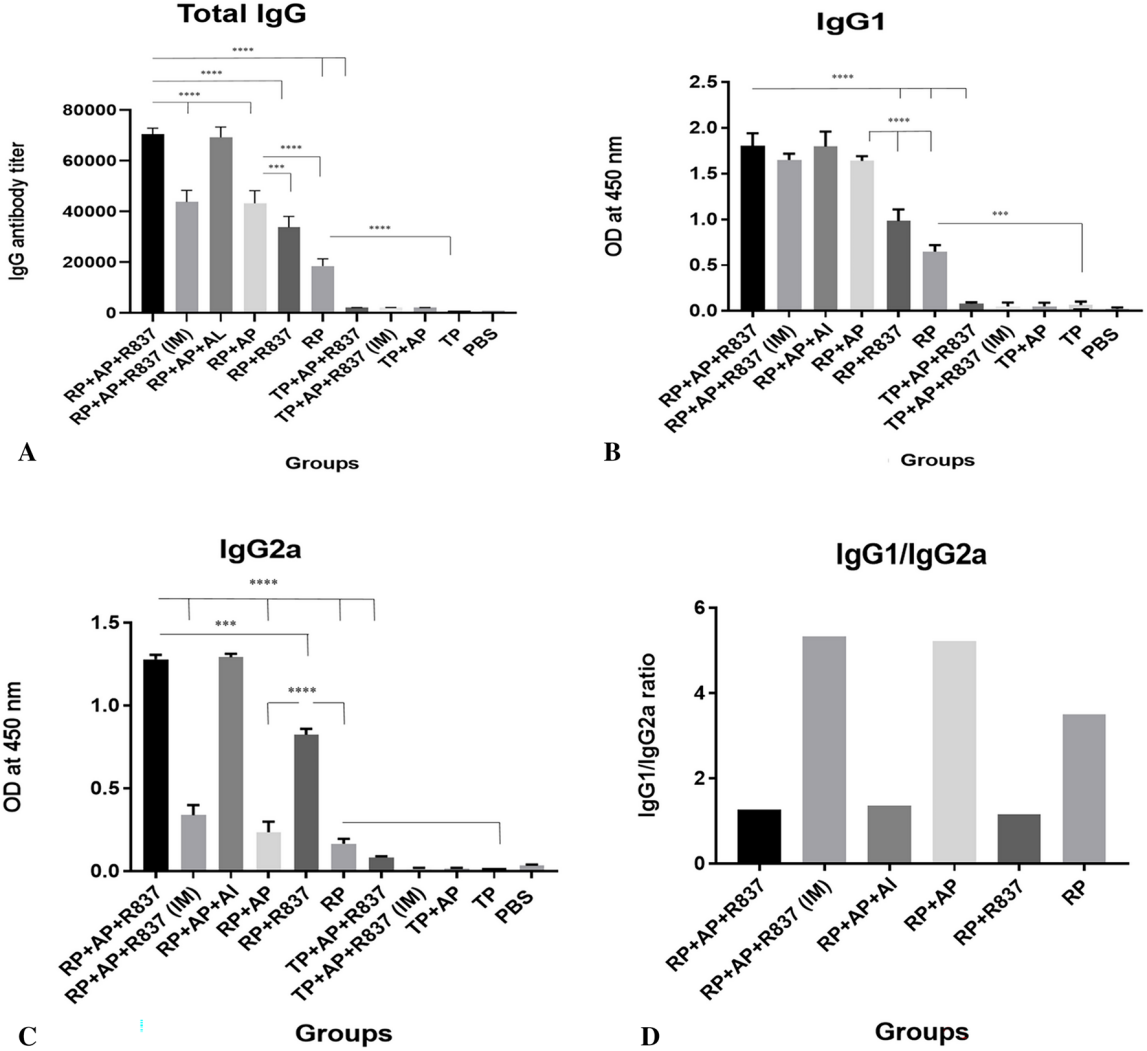
Assessment of humoral immune responses (total and IgG1, IgG2a subclasses) in mice immunized by various formulations. (**A**) Two weeks after last immunization, sera were collected, and total IgG was assessed by ELISA. Antibody titers were calculated as the reciprocal of the latest sera dilution yielding OD_450_ higher than the mean plus 3 SD of the OD values of PBS control group results. (**B**,**C**) IgG1 and IgG2a subclasses were determined at 1/1000 dilution of mice sera. (**D**) Ratio of IgG1/IgG2a subclasses. Data are represented as means ± standard error of means (SEM) of duplicate from 6 mice per group, (*P* < 0.05*, *P* < 0.005**, P < 0.0005*** and *P* < 0.0001****). List of mice groups and abbreviations have been presented in Table 1.

As shown in Fig. 5A–C, assessment of the humoral responses by ELISA indicated that mice immunized by RP-alone or RP-containing formulations (RP + AP + R837, RP + AP, RP + R837, RP) induced significantly higher responses compared to those immunized by any TP-containing formulations (TP + AP + R837, TP + AP, TP + R837, TP) (*p* < *0.0001*). These results indicated the pivotal role of the designed built-in adjuvant within the RP immunogen for induction of anti-RG1 humoral responses. Moreover, the highest level of humoral responses was reached in mice immunized SC by “RP + TLR7 agonist/alum adjuvants” formulations (RP + AP + R837 and RP + AP + AL) compared to other RP-immunized mice groups (*p* < *0.0001*). This observation indicated that TLR7 agonists (R837 or Aldara)/alum adjuvants synergistically enhanced the crucial effect of built-in adjuvants on the induction of anti-HPV RG1 immune responses. Comparing the humoral immune responses (especially IgG2a) for the route of immunization (SC vs. IM) in RP + AP + R837 vaccinated mice indicated that SC was superior to IM injection (*p* < *0.005*). However, there was no significant difference between humoral responses for RP + AP + R837 (SC) and RP + AP + AL (SC) immunization groups, indicating that both TLR7 agonists (R837 and AL) showed similar effects despite different treatment ways. No HPV anti-RG1-specific immune response was detected for control groups (groups immunized by adjuvant mixture or PBS).

As shown in Fig. 5B–D, assessment of IgG subclasses indicated an IgG1/IgG2a ratio between 0.5 to 2.0 for groups receiving TLR7 agonists (R837 or Aldara) by SC immunization (RP + AP + R837, RP + AP + AL or RP + R837) that implies a balanced Th1/Th2 polarization of the immune system. However, in groups deprived of TLR7 agonists either with or without alum adjuvant (RP + AP or RP-alone) or received RP + AP + R837 via IM immunization route, an IgG1/IgG2a ratio above 2.0 was obtained, indicating polarization of lymphocytes to a Th2 phenotype.

#### Immunization by RP + TLR7 agonist/alum adjuvants induced the highest neutralizing antibodies

**Figure 6:**
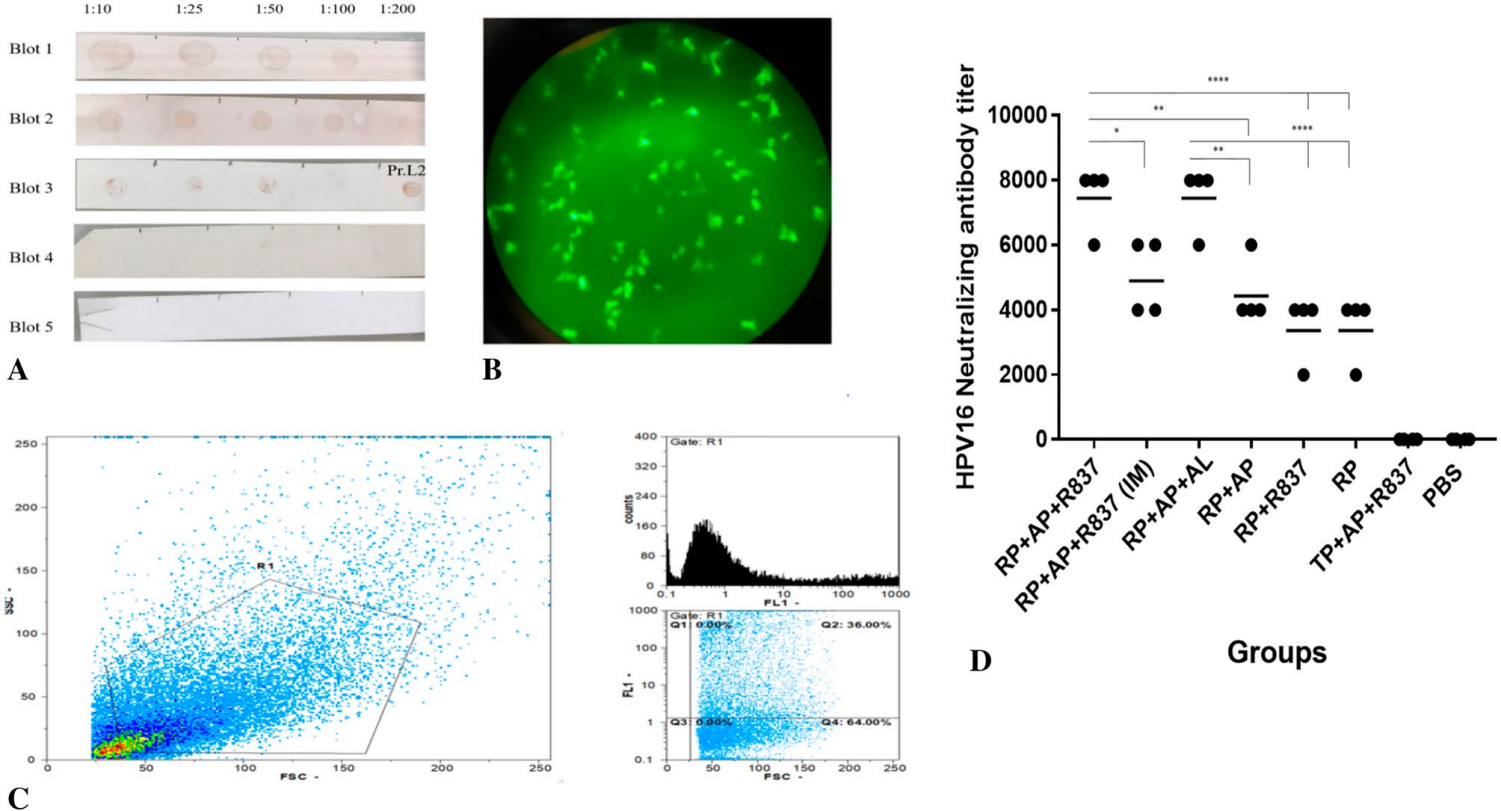
Characterization of the generated HPV16 L1/L2 PsVs and assessment of Neutralization antibodies (nAbs) in mice immunized by various formulations. (**A**) Results obtained from five dot blot experiments using different dilutions of antibodies against L1 and L2 proteins. Blot 1: Gardasil-9 as L1-containing control treated with anti-HPV16 L1 monoclonal antibody. Blot 2: PsVs treated with anti-HPV16 L1 monoclonal antibody. Blot 3: PsVs treated with anti-HPV16 L2 mice sera. Blots 4 and 5: GFP protein as the negative control treated with anti-L1 and anti-L2 antibodies, respectively. (**B**) Fluorescent microscopy of HEK-293LTV cells transduced by the generated HPV16 L1/L2 PsVs for GFP expression 48 h post-transduction. (**C**) Flow cytometry analyses showed that 36% of cells were transduced by PsVs. (**D**) Two weeks after the last immunization, sera were collected, and nAbs titers (starting dilution at 1:1000) against HPV 16 PsVs were detected by in vitro assay. The nAbs titers were expressed as cross-dilution in which 50% of PsVs were neutralized compared to positive control wells. End point titers were plotted and means were reflected as horizontal lines. The data are presented as mean of duplicate ± standard deviation (SD), and (*P* < 0.01*, *P* < 0.001**, P = 0.0002***, *P* < 0.0001****). List of mice groups and abbreviations have been presented in Table 1.

For characterization of HPV16 L1/L2 PsVs, dot blotting was performed (Fig. 6A) that was followed by direct observation of GFP expression of PsVs-transduced HEK-293LTV cells under fluorescence microscope 48 h post-transduction (Fig. 6B). Flow cytometry indicated that the transduction efficiency of HEK-293LTV cells by PsVs (produced at 1:100 dilution) was 36% (Fig. 6C).

As shown in Fig. 6D, assessment of nAbs in immunized mice indicated that mice immunized by RP-alone or RP-containing formulations (RP + AP + R837, RP + AP, RP + R837, RP) induced significantly higher nAbs compared to the mice immunized by TP + AP + R837 that had not detectable neutralizing antibodies. These results, similar to humoral immune responses, again indicated the critical role of the designed built-in adjuvant within the RP immunogen for induction of nAbs. Moreover, the highest level of nAbs was reached in mice immunized SC by “RP + TLR7 agonist/alum adjuvants” formulations (RP + AP + R837 and RP + AP + AL) compared to other RP-immunized mice groups RP + AP (*p* < *0.001*), RP + R837 and RP (*p* < *0.0001*). This observation again indicated that TLR7 agonists (R837 or Aldara)/alum adjuvants synergistically enhance the crucial effect of built-in adjuvants on the induction of nAbs. Similar to results for anti-RG1 humoral responses, comparing the nAbs for the route of immunization (SC *vs*. IM) in RP + AP + R837 vaccinated mice indicated that SC injection resulted in a significantly higher titer of nAbs compared to the IM injection (*p* < *0.01*). Similarly, no significant difference between the level of induced nAbs for RP + AP + R837 (SC) and RP + AP + AL (SC) immunization groups was observed, indicating that both TLR7 agonists (R837 and AL) showed similar effects despite different treatment ways, and nAb titers induced by RP + TL7 agonist/alum adjuvants were significantly higher only when injected subcutaneously. No nAbs were detected for control (groups immunized by adjuvant mixture or PBS) or TP-immunized groups.

#### Immunization by RP + TLR7 agonist/alum adjuvants induced the strongest cellular immune responses

**Figure 7:**
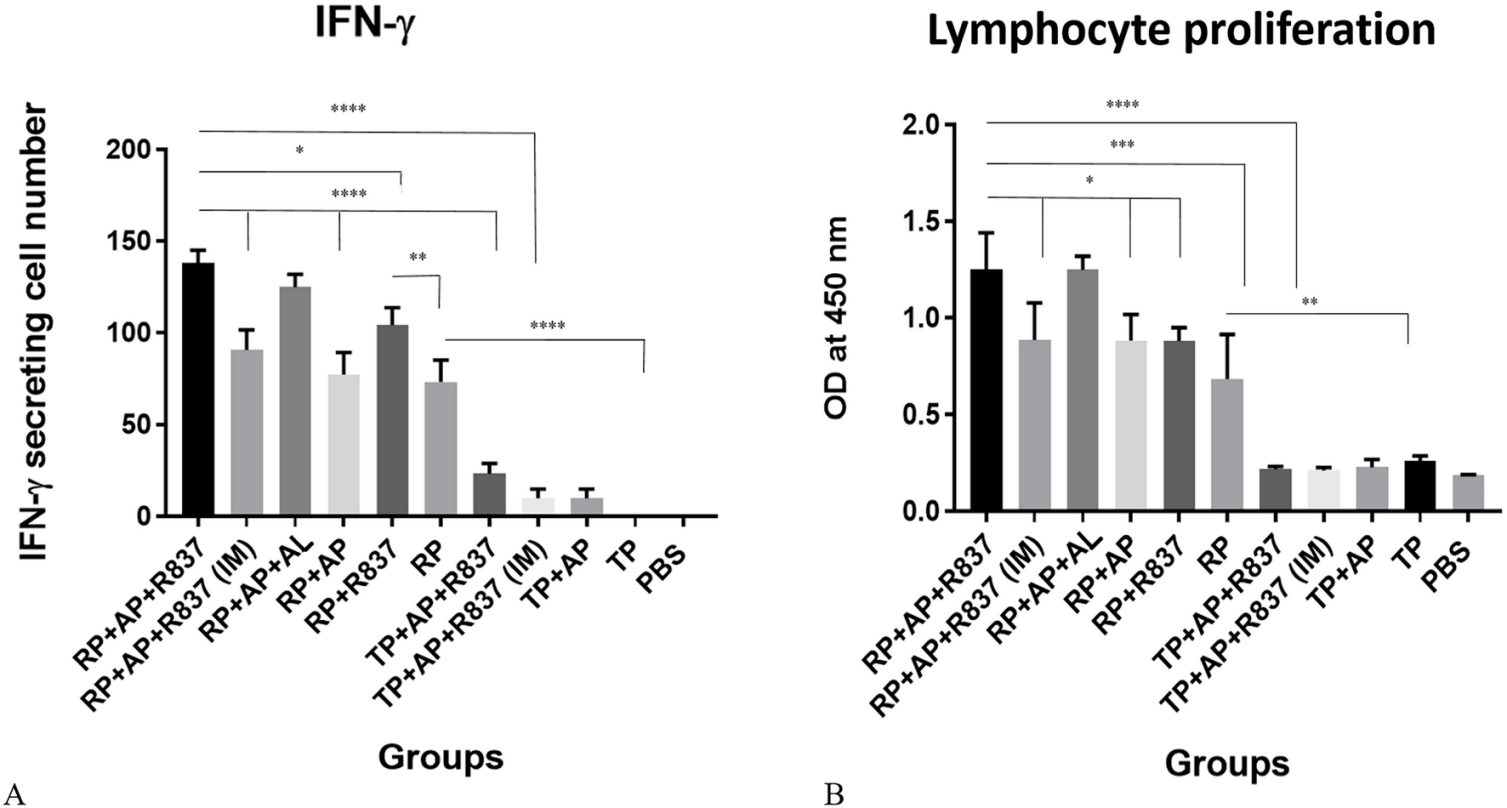
Assessment of IFN-γ secretion (**A**) and Lymphocyte proliferation (**B**) on splenocytes of mice immunized by various formulations. **A)** The level of IFN-γ was determined on splenocytes culture supernatant of immunized mice (n = 6/group) as mean absorbance at 450 nm ± SD for each set of samples. **B)** Lymphocyte proliferation were evaluated in splenocytes of mice per group (n = 6/group) that were stimulated with BrdU method. The data are presented as mean of duplicate of absorbance at 450 nm ± standard deviation SD (*P* < 0.05*, *P* < 0.005**, P < 0.0005*** and *P* < 0.0001****). List of mice groups and abbreviations have been presented in Table 1.

Assessment of IFN-γ secretion and cellular proliferation as two parameters generally presenting the induction of cellular responses showed that mice immunized by RP-alone or RP-containing formulations (RP + AP + R837, RP + AP, RP + R837, RP) induced significantly higher IFN-γ secretion and cellular proliferation compared to TP-immunized groups or controls (Fig. 7). These results, similar to Ab-related responses, again indicated the pivotal role of the built-in adjuvant within the RP immunogen for induction of cellular responses. Similarly, the highest level of IFN-γ secretion and cellular proliferation was reached in mice immunized SC by “RP + TLR7 agonist/alum adjuvants” formulations (RP + AP + R837 and RP + AP + AL) compared to other RP-immunized mice groups for IFN-γ secretion RP + AP, and RP (*p* < *0.0005)* and RP + R837 (*p* < *0.05)*, for cellular proliferation RP + AL, RP + R837 and RP (*P* < *0.0005)*, RP + AP and RP + R837 (*p* < *0.05*). This observation indicated that TLR7 agonists (R837 or Aldara)/alum adjuvants synergistically enhanced the crucial effect of built-in adjuvants on the induction of cellular responses, too. Similar to the results of Ab responses, comparing the cellular responses for the route of immunization (SC *vs*. IM) in RP + AP + R837 vaccinated mice indicated that SC was superior to IM injection (for IFN-γ secretion *p* < *0.001* and for cellular proliferation *p* < *0.05*). Similarly, no significant difference between the level of induced cellular responses for RP + AP + R837 (SC) and RP + AP + AL (SC) immunization groups was observed, indicating that both TLR7 agonists (R837 and AL) showed similar effects despite different treatment ways. No significant cellular responses were detected for the control groups immunized by adjuvant mixture alone or PBS or TP-immunized groups.

## Discussion

In the present study, by extensive in silico analyses, we first showed that an RP-containing built-in-adjuvant harboring TLR5/TLR4 agonists, TT-P2 and HPVL2 RG1 had proper physicochemical, immunogenic and safety profiles as an immunogenic peptide as well as effective interaction with corresponding TLRs. Secondly, by in vitro analyses, we showed stable expression and native purification of the endotoxin-free *E. coli*-derived RP for immunization studies. Finally, by in vivo studies in mice, we showed a crucial role for the built-in-adjuvant in eliciting anti-HPV RG-1 immune responses in immunized mice. We further showed that the combination of TLR7 agonist/alum adjuvants synergistically enhanced the built-in anti-HPV RG-1 immune responses. Moreover, we showed that while the built-in adjuvant elicited Th2-polarized anti-HPV RG-1 immune responses, the combination of a TLR7 agonist changed the polarization to a balanced Th1/Th2 immune response.

Results of in silico analyses (Table 1 and Fig. S1) predicted high stability with an instability index of 35.84 and higher aliphatic indices of 69.63 for the designed RP that is in agreement with the abundance of α-helix and coiled regions in the secondary structure (Fig. S1). These results, in accordance with previous reports, could indicate the importance of the abundant α-helix and coiled regions in the secondary structure of the designed immunogen for proper stability and solubility^14,19^. We further analyzed the interaction between the Entolimod (flagellin)’s D1 domain within RP and TLR5 by molecular docking. Our results (Figs. 3A and 4A), in agreement with prior reports^24,25^, predicted the involvement of the D1 domain region (QRVRELSV) in the interaction of flagellin with the TLR5, and confirmed the role of hydrogen bonds in this interaction. Similarly, analyses by molecular docking indicated the interaction of RS09 within RP and TLR4 via hydrophobic bonds between residues of RS09 and MD2 (co-receptor of TLR4) (Figs. 3B and 4B), which has been previously proposed for that of the LPS and TLR4^26^. In addition, as shown in Table 1, in silico analyses predicted that RP is a non-allergen and non-toxic antigen, and soluble when expressed in *E. coli*. Evaluation of the endotoxin level of the purified RP also indicated values less than 0.01 EU/ml, which was appropriate for the aim of the immunization. Accordingly, evaluation of the local injection sites for any adverse reactions indicated mild and temporary irritations and swellings in immunized mice. Additionally, based on guidelines of the ethical committee of Pasteur Institute of Iran, body weight and food/water consumption were determined weekly, and mice behavior and appearance were observed every other day, and no abnormality was detected. However, detailed safety and toxicity studies incorporating body function analysis through blood and urine factors and histopathologic studies were set to be performed in future “preclinical phase investigation” with dedicated mouse and rat groups.

Expression of 1206 bp DNA sequence encoding RP in *E. coli* BL21 (DE3) resulted in a 46 kDa soluble recombinant protein, which was in agreement with the expected/calculated MW for RP and confirmed the in silico analyses results for the stability/solubility of the RP in the bacterial expression system (Fig. S4A, Fig. 4B). The observed stability of RP which was consistent with a prior report on the expression of RG1 epitope concatemers incorporated into flagellin^7^ was an important achievement in our study compared to the unstable expression of other concatenated RG1-encoding peptides^6,27^.

As shown in Fig. 5A, total IgG levels for immunization by RP-alone were significantly higher than TP groups even in formulation with external adjuvants, indicating the pivotal role of the incorporated built-in adjuvants in eliciting anti-RG1 immune responses. This result is in accordance with a prior study that showed the incapability of immunization with 3 × RG1 peptides formulated with MF59 + poly I:C adjuvant for eliciting anti-RG1 nAbs^6^. Moreover, our result was in agreement with a prior study reporting the critical role of the incorporated built-in flagellin for induction of the serum and mucosal nAbs against fused HPV L2 protein^28^. However, as shown in Fig. 5A, RP + R837 immunization was even superior in the induction of IgG compared to that of the RP-alone group (*p* < *0.005*), indicating the synergistic effect of the built-in adjuvants within RP with TLR7 agonist (Imiquimod) in the induction of the immune responses. Our result was also in accordance with prior reports on the synergistic effect of co-administered TLR4 and TLR7/8 agonists in the enhancement of humoral immunity^29^. Of note, there was further enhancement of humoral responses for RP + AP + R837 (SC injection) group compared to the RP + R837 group (*p* < *0.0005*), which indicated the combined synergistic effects between TLR7 agonist (Imiquimod) and alum adjuvant with built-in adjuvants within RP (Fig. 5A). In agreement with our result, recently synergistic effect of alum adjuvant and a built-in TLR7 agonist for anti-tumor immune responses has been reported^22^. It was previously suggested that the observed synergistic effect might be due to the enhancement of the absorption of aluminum hydroxide by TLR7 agonist^30^. Another point of interest is the total IgG levels for the RP + AP + R837 (SC injection) group, which were not significantly different from that of the RP + AP + AL group. Additionally, this result showed that the administration of 5 mg topical Imiquimod (Aldara) had not essentially increased TLR7 stimulation compared to the injection of 5 μg of soluble form of Imiquimod (R837) (Fig. 5A). Although our result might be contrary to a prior report that suggested better performance of the topical application of Imiquimod compared to injection of the R837 for DC maturation and CD8^+^ T cell cross-priming^31^, our designed built-in adjuvant within RP might have synergistically and equally normalized the immune enhancements of both TLR7 agonists (Aldara and R837). This could be another advantage for the RP when considering both the higher price of “Aldara skin cream” compared to R837, and also the complexity of topical application for general vaccination goals. A further point anticipated from Fig. 5A is the better performance of SC injection compared to IM injection for RP + AP + R837 immunization (*p* < *0.005*). Indeed, IgG levels for IM injection of RP + AP + R837 were not significantly different from the RP + AP group, while it was significantly higher than that of the RP + R837 group (Fig. 5A). This result indicated the negligible adjuvant role of the R837 for IM immunization compared to that of the SC in the induction of Abs. Our result was in accordance with another prior study that reported the superiority of the SC immunization for the adjuvant effect of a TLR7 agonist on induction of Abs to factor IX^32^ and implies the importance of the administration route for the vaccine adjuvant as previously suggested^33^. In fact, the superiority of the SC administration route for imiquimod might be well-anticipated while considering its mechanism of action that involves cytokine induction in the skin^34^.

To gain an insight into the polarization of the lymphocytes immunized by different immunogen formulations, IgG subclasses were assessed (Fig. 5B–D). It is known that IgG1/IgG2a ratio between 0.5 and 2.0 implies a balanced Th1/Th2 polarization, while that of below/equal to 0.5 or above/equal to 2.0 indicates Th1 or Th2 polarizations, respectively^35^. Our results indicated a balanced Th1/Th2 polarization for groups receiving TLR7 agonists (R837 or Aldara) by SC immunization (RP + AP + R837, RP + AP + AL or RP + R837). However, in groups deprived of TLR7 agonists (RP + AP or RP-alone) or those received TLR7 agonists via IM immunization (RP + AP + R837), a Th2-biased immunization was raised. These results, in parallel with our results of total IgG (Fig. 5A) and prior studies, indicated the superiority of the SC immunization for the adjuvant effect of the TLR7 agonist on induction of Abs^32^ and implied the importance of the administration route for the vaccine adjuvant^33^ which is well-adapted to the skin-based mechanism of cytokine induction in these adjuvants^34^. Our results are also in accordance with prior suggestions on the potential role of TLR7 agonists for enhanced activation of Th1 and Th2 responses^36^. Of note, it is already shown that the combination of TLR4 and TLR7 agonists results in a balanced Th1/Th2-type immune response for different antigens^37,38^. Therefore, it is possible that interaction of the TLR4 ligand in the RP construct and TLR7 agonist in our formulations might have shown a similar effect for this balanced Th1/Th2 polarization. On the other hand, the Th2-polarized responses in the RP-alone group (i.e. deprived of adjuvants) might be due to the effect of the built-in adjuvant within RP, which is in accordance with prior studies for activation of the NF-κB signaling pathway towards Th2-type by other built-in adjuvants harboring similar components^39^. This characteristic might be enhanced for RP + AP immunized group due to the use of alum adjuvant, which is well-known for enhancing the Th2 type polarization. However, the adjuvanticity of TLR7 agonist apparently dominated that of the alum in SC immunization as shown for RP + AP + R837 and RP + AP + AL immunized groups.

Beside humoral responses and nAbs, cellular immune responses have been shown to play essential roles in protection against HPV infection^39^. Accordingly, we evaluated the RP-based immunogens for induction of cellular immune responses. As shown in Fig. 7, IFN-γ secretion and lymphocyte proliferation profiles, in agreement with IgG1/IgG2 ratios (Fig. 5D), indicated higher values for SC immunized-TLR7 agonists receiving groups (RP + AP + R837, RP + AP + AL or RP + R837) compared to those showing Th2 polarization (IM immunized-RP + AP + R837, RP + AP and RP-alone). These results, in accordance with prior studies, argue the same discussions provided for the IgG subclasses^32,37,38^ and induction of both humoral and cellular responses against RG-1 by use of the built-in adjuvants^38^. It should be emphasized that the RP-alone group had an impressively higher IFN-γ cytokine secretion and lymphocyte proliferation compared to that of the TP and PBS groups (*p* < *0.0001*). This result indicated once more the pivotal role of the designed built-in adjuvant for inducing anti-RG1 cellular immune responses by RP immunization (Fig. 7) as well as that of the humoral responses (Fig. 5A).

As shown in Fig. 6D, the results of induced nAbs and HPV 16 PsVs neutralization assay was compatible with the total IgG response (Fig. 5A). Indeed, considering the titers of the elicited nAbs by each immunized mice group showed that immunization by RP alone elicited titers up to 4000 while for that of the RP + AP + R837 (SC injection) or RP + AP + AL, the titers reached to 8000 (Fig. 6D). It was previously suggested that nAb titers around/below 4000 induced by RG-1 immunization were enough to neutralize HPV PsVs^6,40^. Therefore, immunization by RP alone or in combination with (R837 or Aldara)/alum adjuvants might be promising for protection against HPV infection. Of note, the elicited titers of nAbs by RP-formulated immunogens in our study (4000–8000) were comparable with that of the prior reports on RG-1 tandems formulated with either Montanide^40^ or TLR2 agonist^6^ adjuvants that elicited less than 4000 and 8000 titers, respectively. However, it should be noted that in these prior reports, different mouse strains; PsVs production and neutralization assay protocols were used and thus a sharp conclusion could not be drawn from comparing their results and ours. As shown in Fig. 6D, results of induced nAbs and HPV 16 PsVs neutralization once more indicated that TLR7 agonists (R837 or Aldara)/alum adjuvants synergistically enhanced the crucial effect of the built-in adjuvant (TLR5/4 agonists/TT-P2) in RP for induction of nAbs. This result is in agreement with a prior study on the synergistic effect of TLR4 and TLR7 agonists for enhancement of the immunity against a recombinant influenza virus hemagglutinin (HA) to protect against the infection^37^. It should be noted that for the neutralization assay, we only tested HPV 16 PsVs and not the other HPV types since it was not the aim of this study to show the cross-protection capability of the induced nAbs by RG-1 immunization. Prior studies have also well-documented the pan-genomic protection of anti-RG-1 nAbs^4–6,40^.

Finally, as a limitation of our study, it should be noted that we did not have any direct result on the contributing component(s) of built-in adjuvant (TLR5/4 agonists/TT-P2) that interact with TLR7 agonists for enhancement of the anti-RG1 humoral and cellular responses. Whether TLR5 or TLR4 agonists or TT-P2 or some or all components were involved should be addressed in future studies by constructing separate immunogens for various components. However, anticipating from prior reports, TLR4 agonists might have a crucial role in interaction with TLR7 agonists^29,37,41^.

As a promising competitive strategy to elicit cross-type neutralizing antibodies, the *E.coli*-derived engineered chimeric HPV particles that have been synthesized through loop swapping approach have recently been introduced^42,43^. Although the results of their immunogenicity have been encouraging, antigen swapping even in case of being homologous is still under study, and L2-based vaccines, while being studied as a non-VLP based vaccine candidate, could be potentially considered as less-complex proteins to be produced and purified.

Taken together, to the best of our knowledge, we provided the first report consisting of both solid and joint in silico and in vivo data to show the crucial role of a properly designed built-in adjuvant harboring TLR agonists and TT-P2 on anti-RG1 immune responses. In addition, our data for the first time showed the synergistic effect of TLR7 agonist/alum to enhance the adjuvanticity of built-in “TLR4/5 agonists/(TT)-P2” for induction of a robust anti-HPV RG1 immune response. Our results further indicated the role of TLR7 agonist for changing the Th2-polarization that was otherwise elicited by the designed built-in adjuvants to a balanced Th1/Th2 anti-HPV RG-1 response. The result of this study might help to achieve a promising vaccine formulation for the induction of anti-HPV L2 RG1 immune responses that otherwise might result in the production of an inexpensive pan-genomic and cross-reactive HPV vaccine which should be investigated in future preclinical/clinical phase studies on this immunogen.

## Methods

### In silico analyses

#### Retrieval of sequences and design of the rejoined immunogenic peptide (RP)

The selected epitope and peptide sequences used for the construction of the rejoined immunogenic-peptide (hereafter “RP” standing for Rejoined Peptides) that contained the dual 3 × tandem repeats of the RG1 epitope + TLR5/4 built-in-adjuvants + tetanus toxoid epitope “d3RG-T5-TT-T4” in tandem) have been shown in Table S1. The sequences of the HPV16 RG-1 epitope (Accession No. P03107) and the tetanus toxoid P2 epitope (TT-P2; containing 15 residues; Accession No. P04958.2) were retrieved from the National Center for Biotechnology Information (NCBI) database (https://www.ncbi.nlm.nih.gov/)^44^. The Entolimod (the TLR5 agonist) sequence (Accession No. KEGG DRUG: D10368), as a derivate of Salmonella flagellin, was obtained from KEGG online database (https://www.genome.jp/)^45^. The previously reported sequences of the short peptide, RS09 (the TLR4 agonist)^16^, the 6xHis-tag^46^ and the flexible linker (GGGS)_3_^47^ were obtained from valid data sources. Mouse TLR4 (UniProt ID. Q9QUK6) and mouse TLR5 (UniProt ID. Q9JLF7) sequences, used for molecular docking analyses, were retrieved from the UniProt database (https://www.uniprot.org/)^48^.

For designing the construct, as shown in Fig. 1, the three tandem copies of the HPV16 RG-1 epitope were incorporated twice in the positions that potentially can make them highly accessible for antibody induction^49^. Accordingly, N- and C- termini of D1 domain (D1^N^ and D1^C^) of flagellin that are expected to activate the NF-κB signaling pathway after binding to TLR5, were embedded in the construct^24^. The D1^c^ domain of the flagellin was linked to the TT-P2 that has also been reported previously^10^. Finally, the RS09 and His-tag encoding sequences were fused to the C-terminus of the sequence (Fig. 1), and thee flexible linkers (GGGS)_3_ were used for joining the segments. The final 436-residue sequence of the RP, shown in Table S1, was used as input for bioinformatics analyses.

#### Evaluation of the physicochemical, immunogenic and safety profiles of the RP

The physicochemical properties of the RP, such as its molecular weight (MW), amino acid composition, theoretical pI, instability index, aliphatic index, and grand average of hydropathicity (GRAVY), were determined by ProtParam tool (http://web.expasy.org/protparam/)^50^. Also, the antigenicity, the potential allergenic characteristics and toxicity of the RP were investigated by the VaxiJenv2.0 (http://www.ddg-pharmfac.net/vaxijen/VaxiJen/VaxiJen.html)^51^, AllergenFP v.1.0 (http://ddgpharmfac.net/AllergenFP/)^52^), and the Toxinpred (http://crdd.osdd.net/raghava/toxinpred) servers^53^, respectively. To predict the solubility of the protein following overexpression in *E. coli* and to distinguish between soluble and insoluble proteins based on the physicochemical properties, the sequence of the RP was evaluated by the Solpro (http://scratch.proteomics.ics.uci.edu/)^54^ and ccSOL (http://www.tartaglialab.com/) servers^55^, respectively.

#### Secondary and 3D structure analyses of the RP

The secondary structure of the RP was predicted by the SOPMA (Self-Optimized Prediction Method with Alignment) server (http://npsa-pbil.ibcp.fr). This tool predicts solvent accessibility, transmembrane helices, globular regions, bend regions, random coil, and coiled-coil regions of the protein^56^.

For 3D structure modeling of the construct, the I-TASSER server (http://zhanglab.ccmb.med.umich.edu/ITASSER/) was used, and the 3D modeling results were valued as a C-score: a reliability score for evaluating the quality of the models predicted by I-TASSER. The C-score is usually in the range of − 5 to 2, where a higher value of the C-score indicates a model with high confidence^57^.

To improve the quality of the I-TASSER-derived model, the 3Drefine server (http://sysbio.rnet.missouri.edu/3Drefine/) was used. This tool refines the protein structure stability with hydrogen bonding network optimization, and minimization of atomic energy^58^ that can be lastly visualized by the Pymol viewer. Finally, for model validation analyses, several web-based servers were used, including (i) ProSA (https://prosa.services.came.sbg.ac.at/prosa.php) that calculates an overall quality score (Z-score) for a specific input structure^59^, (ii) the ERRAT (http://services.mbi.ucla.edu/ERRAT/) that analyzes the statistics of non-bonded interactions between different atom types^60^, and (iii) the RAMPAGE (http://mordred.bioc.cam.ac.uk/~rapper/rampage.php) that shows the preferred areas^61^.

#### Protein–protein flexible docking

The interaction of the incorporated flagellin D1 domain and RS09 in the RP construct with TLR5 and TLR4/MD2 (a co-receptor of TLR4), respectively, were docked using the SwarmDock server (https://bmm.crick.ac.uk/~svc-bmm-SwarmDock/) that carries out the flexible modeling of protein–protein interactions^62^. For the sake of comparing, the interaction of unincorporated flagellin and RS09 with TLR5 (PDB ID: 3V47) and TLR4/MD2 (PDB ID: 3VQ2, after substituting LPS) were also docked, respectively as controls. Finally, the molecular interaction between the best-docked complexes, retrieved from molecular docking studies, was analyzed using Dimplot in LIGPLOT software^63^. This program produces a plot, including hydrophobic interaction and hydrogen-bonding pattern between receptor and ligand.

### In vitro studies

#### Expression and characterization of the proteins and peptides

The RP-encoding DNA sequence was synthesized and subcloned into the pET28a vector between *Nco*I and *Xho*I restriction sites (Biomatik, Canada) for expression in *E. coli* BL21 (DE3). Protein expression was induced by adding 1 mM IPTG (Isopropyl-β-1-thiogalactopyranoside) (Sigma, UK) at 37 °C. After four hours of incubation, the cell pellet was prepared for the purification step. The protein expression was confirmed by SDS-PAGE and Western blot using anti-6X His tag antibody (Abcam, UK) according to the standard protocol^64^. The recombinant protein was purified in the native conditions on the Ni–NTA agarose column based on the producer’s manual (Qiagen, Germany)^65^, and protein concentration was determined by the Bradford method^66^.

The solubility of the expressed protein was measured based on the percentage of the protein extracted from cell lysate vs protein trapped in cell debris. Additionally, the endotoxin level of the purified recombinant RP protein was evaluated by the chromogenic Limulus amoebocyte lysate (LAL) test (BioWhittaker, UK) according to the manufacturer protocol.

Another protein harboring only the dual 3 × tandem repeats of the RG1 epitope without incorporation of the built-in TLR4/5 agonists and TTP2; hereafter: TP (Triple peptides) (kindly provided by Dr. Motevali)^40^ was used as a control in immunization studies to investigate the effect of the built-in TLR4/5 agonists and TTP2, and also alum/TLR7 adjuvants on the level of anti-RG1-elicited immune responses. Additionally, the HPV16 L2 capsid protein (11–88 aa) (kindly provided by Dr. Motevali)^40^ was used as the coating protein for ELISA to measure the humoral anti-RG1 immune responses.

#### Production of HPV16 L1/L2 pseudoviruses (PsVs)

HPV16 L1/L2 PsVs were generated using the previously described procedures^67^ with minor modifications. In brief, the HPV16 L1h/L2h plasmid expressing the HPV16 capsid proteins (kindly provided by Professor Martin Muller, Germany) together with the pEGFP-N1 reporter plasmid expressing the green fluorescent protein (Addgene, USA) were co-transfected into HEK-293LTV cells using calcium phosphate method^68^. Sixteen hours after transfection, the medium was removed and replaced with fresh and prewarmed medium containing 10% FBS. The cells were investigated for GFP expression by fluorescence microscopy 48 h post-transfection, and were harvested by centrifugation. The cell pellets were then gently resuspended in the cell lysis and pseudovirus maturation buffer containing 0.5% Triton X-100, 5 mM CaCl_2_, 10 mM MgCl_2_, and 25 mM (NH_4_)_2_SO_4_ in PBS and incubated at 37 °C for 24 h. The cell lysate was chilled and centrifuged at 5000×*g* for 10 min^69^. The supernatant was then investigated for PsVs using dot blotting according to the standard protocols^70^ by means of anti-HPV16 L1 monoclonal antibody (SCBT, USA) and the serum of mice immunized with L2 protein (11–88 aa) for detection of L1 and L2 proteins, respectively. The Gardasil-9 commercial vaccine and L2 protein (11–88 aa) were used as the positive controls. Briefly, samples were placed on the nitrocellulose membrane, blocked with bovine serum albumin (BSA) and incubated with the primary antibody, before washing and incubation with HRP-conjugated secondary antibody. The spots were then developed by application of DAB substrate (Sigma, UK). Generation of HPV 16 L1/L2 PsVs were further confirmed by transducing HEK-293LTV cells with pseudoviral stock and direct observation of GFP-expressing cells under a fluorescence microscope. The pseudoviral stock was stored at − 70 °C until further use.

### In vivo studies

#### Mice and immunization

All animals were obtained from the experimental animal center at the Pasteur Institute of Iran, and animal studies were reviewed and approved by ethical committee of the Pasteur Institute of Iran under Ethical Number: IR.PII.REC.1399.078. We also confirm that all methods involving the animals follow the recommendations in the ARRIVE guidelines, and all experiments were performed in accordance with the relevant guidelines and regulations. A total of 72 female BALB/c mice, 6–8 weeks old, were randomly divided into 12 groups (6 per group). As shown in Table 2, ten groups were injected subcutaneously and two groups intramuscularly, three times with the same immunogen formulations on days 0, 28, and 42. Nine groups were injected with 20 μg of either RP or TP alone or in combination with either 5 μg of imiquimod (R837) (Invivogen, USA) or 2% (ratio 1:1) aluminum phosphate (AP) or a mixture of both adjuvants (Table 2). One group was immunized subcutaneously by RP + AP mixture after topical treatment of each mouse with Aldara 5% cream (Meda, Sweden) containing 5 mg of imiquimod as the TLR7 agonist. Mice injected with either PBS or AP + R837 were the control groups. Two weeks after the last injection, blood samples were collected from all experimental groups through retro-orbital bleeding and sera were maintained at − 70 until use.

**Table 2:** Group of mice immunized by different immunogen formulations.

Groups	Injection type	Immunogen*
1	SC	RP + AP + R837
2	IM	RP + AP + R837
3	SC	RP + AP + AL
4	SC	RP + AP
5	SC	RP + R837
6	SC	RP
7	SC	TP + AP + R837
8	IM	TP + AP + R837
9	SC	TP + AP
10	SC	TP
11	SC	PBS/ AP + R837

*RP* (rejoined peptides presenting the complete immunogen harboring the double triple tandem repeats of the RG1 epitope and TLR4/5 built-in-adjuvants and tetanus toxoid); *TP* (triple peptides: Presenting the double triple tandem repeats of the RG1 epitope = 6xRG); R837, AP and AL denote the R837 imiquimod, Alum phosphate and Aldara imiquimod cream adjuvants, respectively. *IM* intramuscular injection, *SC* subcutaneous injection. *PBS* denote phosphate Buffer saline (indicating the control groups either injected by PBS or adjuvant mix “AP + R837” that are deprived of antigens).

*Mice were immunized three times with the same immunogen formulations on days 0, 28, 42.

#### Evaluation of humoral immune responses

The previously described in house-developed ELISA was used to assess the titer of the total IgG and IgG1 and IgG2a subclasses of the immunized mice^40^. To this end, ELISA 96-well plates (Nunc, Denmark) were coated overnight with 6 μg/ml HPV 16 L2 protein (11–88 aa) as described before. The coated plates were blocked with 1% BSA at 4 °C and incubated with the 1:1000 diluted serum of immunized mice from each group separately for 1 h at RT. After the washing steps, wells were incubated for 1 h at 37 °C with goat anti-mouse IgG antibodies HRP-conjugated at dilution of 1:10,000 (Sigma, USA). After the washing steps, wells were incubated for 30 min with TMB substrate (Sigma, USA) and the reaction was stopped with 2 N sulfuric acid. Finally, the optical density of wells was measured at 450 nm by an ELISA reader. IgG1 and IgG2a subclasses were also determined using standard kits (Sigma, UK) according to the manufacturer’s instructions.

#### PsVs-based neutralization assay

Neutralization assay was performed using the generated HPV16 PsVs as previously described^67^ with minor modifications. Briefly, 3 × 10^4^ HEK-293LTV cells were seeded in each well of a 96-well plate using the DMEM (Dulbecco’s modified Eagle’s medium) with high glucose (Gibco, USA) supplemented with 2 mM l-glutamine, 0.1 mM non-essential amino acids, and 10% FBS (Biosera, England). The next day, 1:100 dilutions of PsV was mixed with the pooled sera from each immunized group (in separate wells) at different dilutions starting at 1:1000, and incubated on ice for 1 h before adding to the previously plated HEK-293LTV cells. After 48 h, the cells were trypsinized, and GFP-expressing cells were analyzed by flow cytometry. Neutralization titer was calculated as the highest serum dilution causing more than 50% infection inhibition. Cells treated with only diluted PsV were considered as the negative control.

#### Evaluation of IFN-γ secretion and T cell proliferation

The IFN-γ cytokine was measured by ELISPOT kit according to the manufacturer’s protocol (Mabtech, Sweden). In brief, mice were sacrificed, their spleen were removed, and isolated splenocytes were washed with saline containing 10% FBS and 1% Pen/Strep, and treated with RBC lysis buffer for 8 min at 4 °C. Following centrifugation, 2 × 10^7^ cells/ml were seeded in 24 well plates for 72 h in the presence of either 10 μg/ml L2 protein (11–88 aa) or 5 μg/ml of phytohemagglutinin (PHA) as positive control. After 72 h, the supernatant was harvested to evaluate the secretion of IFNγ by ELISPOT assay.

T cell proliferation was measured using the Bromodeoxyuridine (BrdU) ELISA kit according to the manufacturer’s protocol (Roche, Germany). In brief, after washing steps and treatment of the splenocytes by the same amount of either HPVL2 protein or PHA and incubation for 72 h at 37 °C, BrdU was added to each well and incubated for another 24 h at 37 °C. The next day, the medium was removed, cells were fixed, and anti-BrdU was added to each well. After washing steps and the addition of TMB (Sigma, USA), the reaction was stopped by adding sulfuric acid, and the absorbance at OD_450_ nm was measured.

#### Statistical analysis

One-way ANOVA and Bonferoni multiple comparison were applied to analyze data using GraphPad Prism version 7.03 program (GraphPad Software, San Diego, CA). The data were expressed as mean ± SD, and the *P* < *0.05* was considered statistically significant.

### Supplementary Information

Supplementary Information.

## Data Availability

All data generated or analysed during the study are included in the submitted manuscript and its supplementary information files. The raw data and data that support the findings of this study are available from the corresponding author upon request. There are no restrictions on data availability.
