# Supplementary material for: Alum and a TLR7 agonist combined with built-in TLR4 and 5 agonists synergistically enhance immune responses against HPV RG1 epitope
Source: Sci Rep. 2023 Oct 5;13:16801. doi: 10.1038/s41598-023-43965-3 (PMC10556035; doi:10.1038/s41598-023-43965-3)
Supplement: Supplementary file 1 — Supplementary Information. [file 41598_2023_43965_MOESM1_ESM.docx]

**Supplementary:**

**Table S1.** The selected epitopes and peptides sequences used for construction of the rejoined immunogenic-peptide (RP)

| Amino acid Sequences | Epitopes and peptides* |
| --- | --- |
| QLYKTCKQAGTCPPDIIPKV | RG-1 |
| SNIKGLTQASRNANDGISIAQTTEGALNEINNNLQRVRELSVQATNGTNSDSDLKSIQDEIQQRLEEIDRVSNQTQFNGVKVLSQDNQMKIQVGANDGETITIDLQKIDVKSLGLDGFNVNTLINEDAAAAKKSTANPLASIDSALSKVDAVRSSLGAIQNRFDSAITNLGNTVTNLNSAR | D1 domain |
| QYIKANSKFIGITEL | TT-P2 |
| APPHALS | RS09 |
| HHHHHH | His-tag |
| GGGGS | Flexible Linker |

* The abbreviated names denote the following terms: RG-1: HPV L2 conserved epitope, D1 domain of flagellin as the TLR5 agonist; TT-P2: tetanus toxoid epitope (universal T-cell helper); RS09: the short TLR4 agonist peptide; His-tag: 6xHis tag for facilitating detection and purification of the antigen; Flexible linker: (GGGS)_3_.

**Table S2.** Refinement results of the 3D modeled structure by 3Drefine server

| No. | 3Drefine score | GDT-TS | GDT-HA | RMSD(A) | MolProbity | RWPlus |
| --- | --- | --- | --- | --- | --- | --- |
| 1 | 24475.0 | 0.9983 | 0.9607 | 0.393 | 3.338 | -58787.856199 |
| 2 | 24856.0 | 0.9994 | 0.9677 | 0.361 | 3.322 | -58674.169593 |
| 3 | 25436.4 | 0.9994 | 0.9769 | 0.325 | 3.257 | -58582.071737 |
| 4 | 30674.0 | 1.0000 | 0.9977 | 0.202 | 3.192 | -58508.136838 |
| 5 | 26593.8 | 1.0000 | 0.9873 | 0.278 | 3.207 | -58406.465879 |

The 3Drefine server is able to indicate several parameters by means of various tools, including global distance test (GDT-TS; indicates the distance between the atoms), RMSD (indicates the square deviation of carbon atoms), MolProbity (indicates energy of atoms based on the structure), and RWPlus (indicates the potential energy of atoms based on distance). The measured values for the explained parameters indicated that the 3D structure of the RP is optimal in terms of the network of hydrogen bonds, and the amount of energy at the level of atoms has been minimized.

**Fig. S1.** SOPMA analysis of the secondary structure. Lines in different colors represent different conformational states: Blue: alpha-helix; Green: beta-turn; Red: extended strand, and Purple: random coil. According to the percentages of α-helix, β-turn and random coil, the recombinant protein is a stable and polar protein.

**
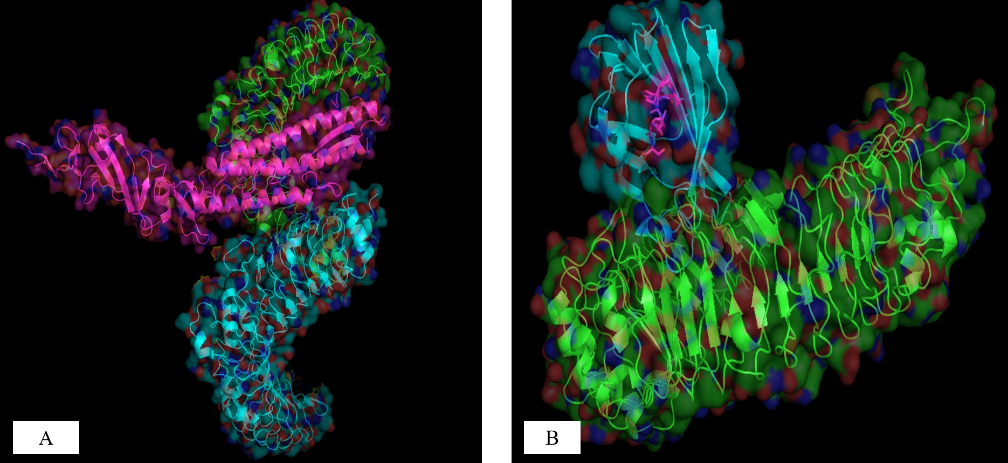
**

**
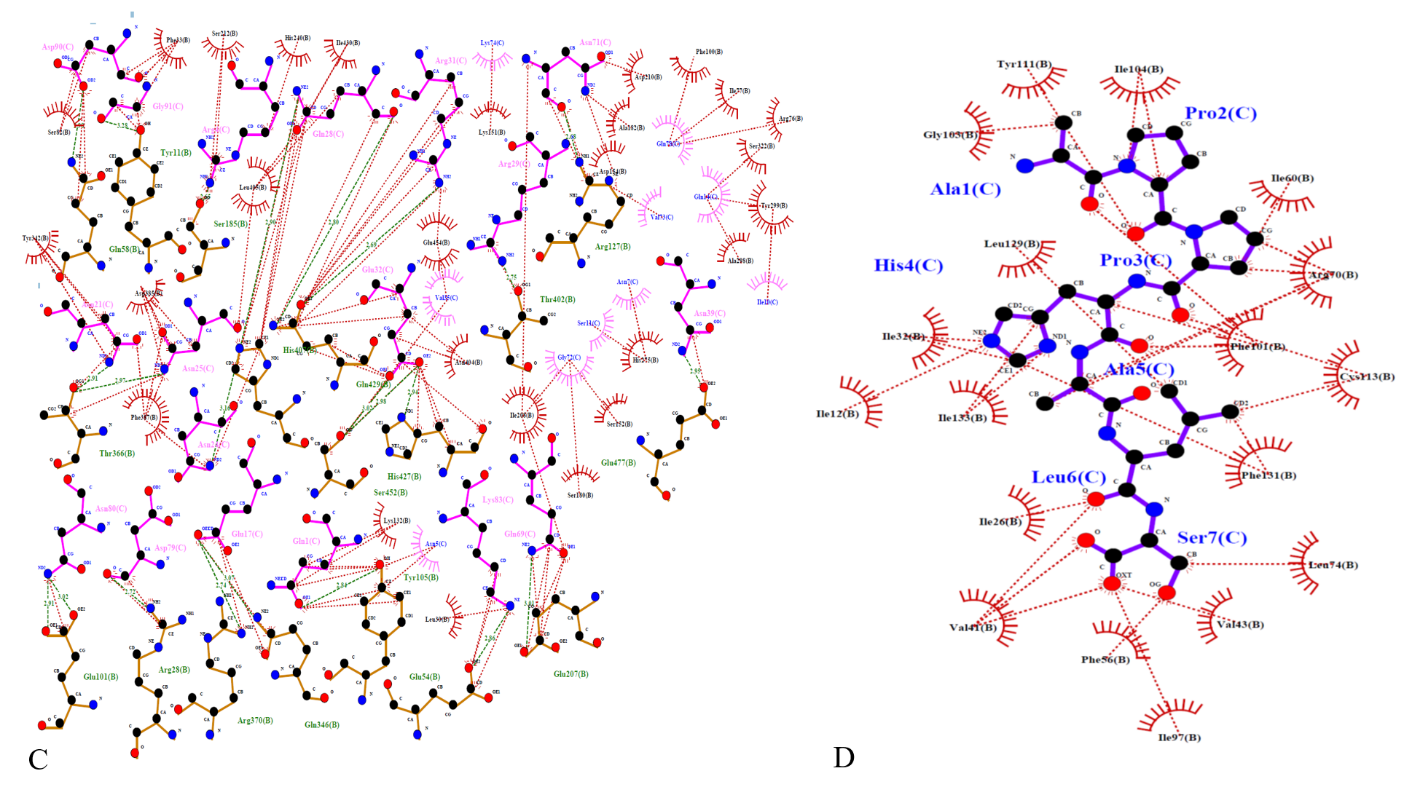
**

**Fig. S2.** The 3D structure of molecular docking simulation between TLR 4 and 5 with their natural ligands (control). A) Molecular docking of natural Salmonella flagellin with TLR5. The pink chain is the natural Salmonella flagellin and the blue and green chains are TLR5. B) Molecular docking of the intact RS09 epitope with TLR4/MD2. The pink chain is the RS09, the blue chain is MD2, and the green chain is TLR4. C) The interactions of Gln 28, Arg 29, Arg 31, and Glu 32 of the conserved QRVRELSV peptide of D1 domain with TLR5 are shown. D) The interactions of Tyr 111, Ile 104, Gly 103, Ile 60, Arg 70, Leu 129, Phe 101, Cys 113, Phe 131, Ile 12, Ile 32, Ile 133, Ile 26, Val 41, Phe 56, Val 43, Leu 74, and Ile 97 of the MD2 with Ala 1, Pro 2, Pro 3, His 4, Ala 5, Leu 6, and Ser 7 of the RS09 are shown. The interaction of RP with TLR4 and TLR5 receptors was in the total energy range of the control groups, and the connections were between the correct amino acid positions with appropriate bonds in the allowed angles according to the control groups.

**
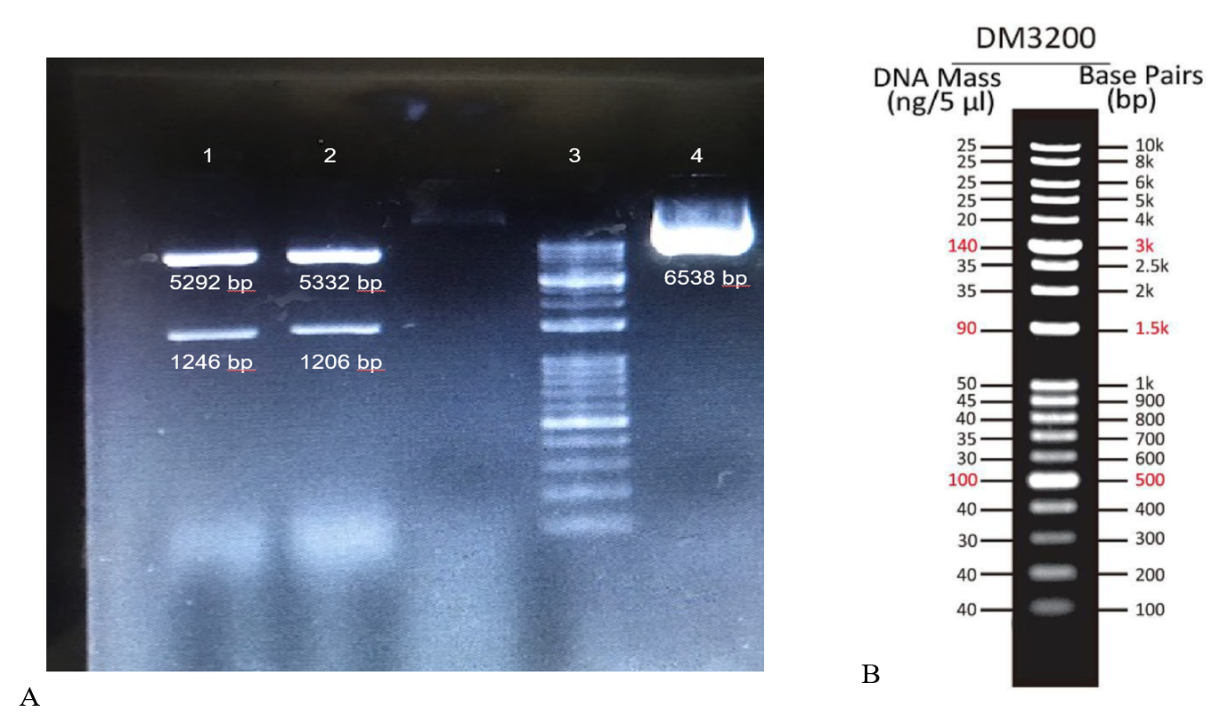
**

**Fig. S3.** Agarose gel electrophoresis/restriction analysis of the pET28a vector harboring 1301 bp RP-encoded DNA. **A)** Lane 1: Digestion with *Xba*I and *Xho*I enzymes resulted in two fragments of 5292 bp and 1246 bp. Lane 2: Digestion with *Nco*I and *Xho*I enzymes resulted in two fragments of 5332 bp and 1206 bp. Lane 3: DNA marker (SMOBIO 10kb, Taiwan). Lane 4: Undigested pET28a vector harboring 1301 bp RP-encoded DNA. **B)** DNA marker DM3200 (SMOBIO 10kb, Taiwan).

**
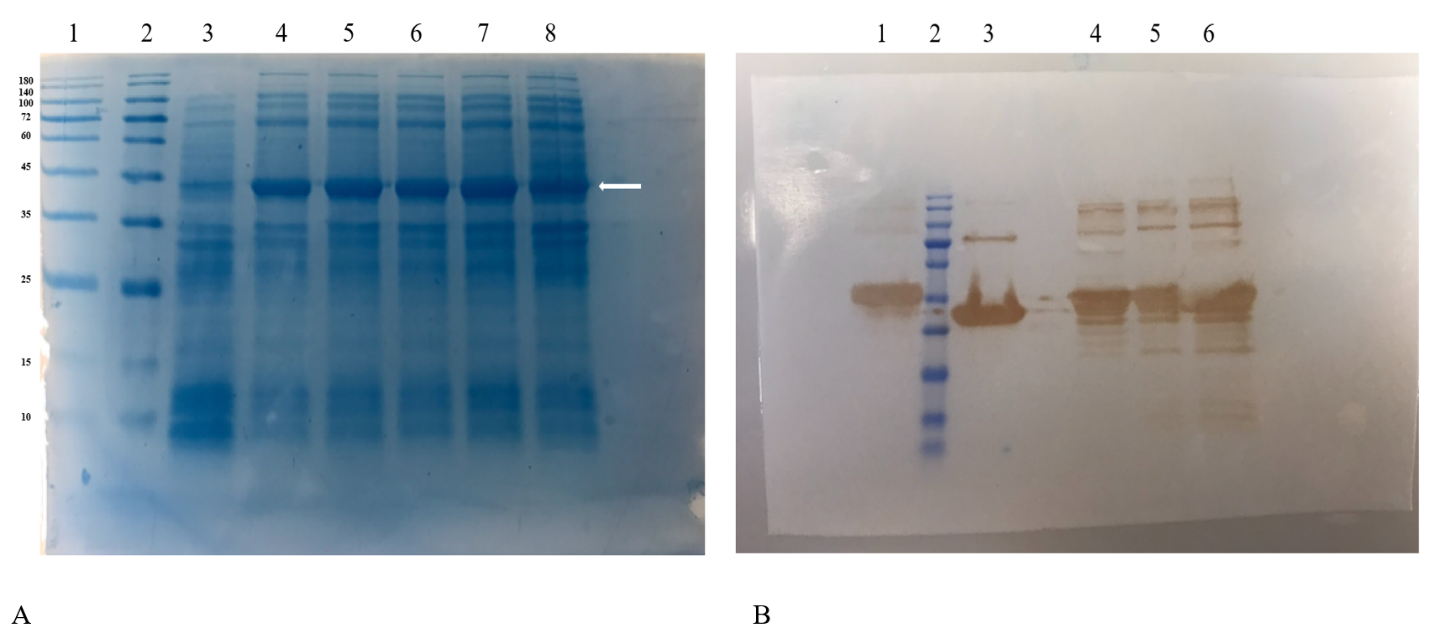
Fig. S4.** Characterization of the *E. coli*-derived RP protein by SDS-PAGE and WB analyses. **A)** SDS-PAGE analysis before and after induction of pET28a-RP-harboring *E. coli* with IPTG revealed a protein band of approximately 46 kDa corresponding to the expressed RP. Lanes 1 and 2: Molecular weight marker (SMOBIO, Taiwan). Lane 3: Un-induced cell lysates of the *E. coli* BL-21(DE3) cells harboring pET28a-RP. Lanes 4-8: Cell lysates of *E .coli* cells harboring pET28a-RP after 3, 4, 5, 6, 16 hours induction with IPTG. **B)** Western blotting results for the expressed RP. Lane 1: Purified RP protein, Lane 2: Molecular weight marker. Lane 3: Purified HPV16 L2 (11-88 aa) protein (used in ELISA for analyses of immunized mice sera). Lane 4-6: Un-purified RP protein expressed after 4 h of induction.


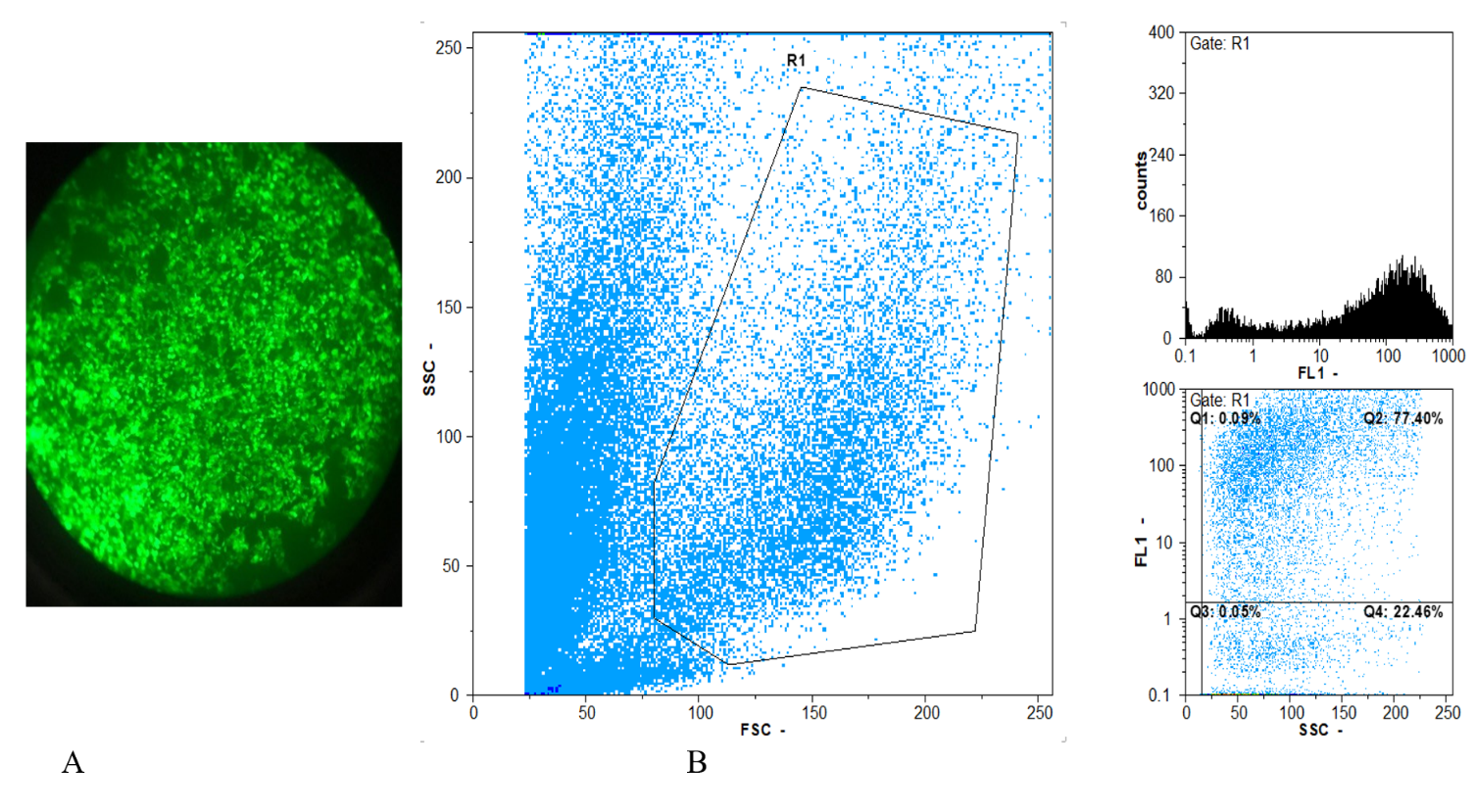
 **Fig. S5.** Expression of GFP in the transfected HEK-293LTV cells. Expression of the HPV16 pseudovirus with GFP as reporter gene after 48 h of transfection. **A)** Transfected HEK-293LTV cells were analyzed by fluorescent microscopy. **B)** Transfected cells expressed the GFP as determined by flow cytometry.
